# Enhancement of the Controlled-Release Properties of Chitosan Membranes by Crosslinking with Suberoyl Chloride

**DOI:** 10.3390/molecules18067239

**Published:** 2013-06-19

**Authors:** Chao Chen, Zideng Gao, Xiaoyun Qiu, Shuwen Hu

**Affiliations:** Department of Environmental Sciences & Engineering, College of Resources & Environmental Sciences, China Agricultural University, Beijing 100193, China; E-Mails: chengchao-0610@163.com (C.C.); gzdsch@163.com (Z.G.); qiuxiaoyun@live.cn (X.Q.)

**Keywords:** chitosan, crosslink, *N*-phthaloylation, hydrophobicity, controlled release

## Abstract

A novel crosslinking agent, suberoyl chloride, was used to crosslink *N*-phthaloyl acylated chitosan and improves the properties of chitosan membranes. Membranes with different crosslinking degrees were synthesized. The derivatives were characterized by Fourier transform infrared spectroscopy and ^13^C solid state nuclear magnetic resonance spectroscopy, which indicated that the crosslinking degrees ranged from 0 to 7.4%. The permeabilities of various plant nutrients, including macroelements (N, P, K), microelements (Zn^2+^ and Cu^2+^), and a plant growth regulator (naphthylacetic acid), were varied by moderate changes in crosslinking degree, indicating that the controlled-release properties can be regulated in this way. The film-forming ability of native chitosan was maintained, whilst mechanical properties, hydrophobicity and controlled permeability were improved. These dramatic improvements occurred with a small amount of added suberoyl chloride; excessive crosslinking led to membranes with unwanted poor permeability. Thus, both the mechanical properties and permeability of the crosslinked membrane can be optimized.

## 1. Introduction

Controlled-release materials are promising for medical science [1,2,3], biotechnology [4,5], and particularly for agricultural formulations such as fertilizers [6,7], herbicides [8,9], pesticides [10,11], and plant-growth regulators (PGRs) [12]. Controlled-release formulations (CRFs) for agrochemicals are able to address problems such as leaching, volatilization, and surface migration because of their sustained-release properties and better performance than conventional formulations, even at lower dosages [13]. Membranes [14,15], fibers [16], and hydrogels [17,18] are the most developed platforms for targeted delivery. Of these, membranes are the most important formulation types for controlled-release fertilizers since they allow manipulation of the rate and duration of nutrient release [19]. There are some commercialized products, such as polyolefin-, polyurethane-, and alkyd resin-coated fertilizers, but these coating materials do not degrade well in soil, and the accumulation of non-degradable polymers in soil will cause soil structure damage, and is contradictory with the purposes of sustainable agriculture. Thus finding some degradable coating materials for controlled release fertilizer is essential [20].

Chitosan (CS) is a natural polysaccharide produced by deacetylation of chitin, the second most abundant polysaccharide on Earth. This biopolymer is biocompatible, biodegradable, and non-toxic. It has been widely adopted for the manufacture of controlled-release materials in the food, drug, biochemical, and agricultural areas [21]. Wu also applied this system in controlled-release fertilizer studies [22]. 

Although chitosan is an attractive biomacromolecule, it is reasonably biodegradable in an outdoor environment, and erosion of the fertilizer membrane shortens the duration of the fertilizer effectiveness. Additionally, chitosan is insoluble in most common organic solvents and water, which greatly limits its applications in a variety of fields. Furthermore, because of its many inter- and intra-molecular hydrogen-bonding interactions, chitosan has a rigid and brittle nature, which adversely affects its processability, and this has severely limited the development of chitosan membranes [23,24]. However, chitosan contains hydroxyl groups and highly reactive amino groups, and has been successfully modified by several different chemical reactions, including *N*-alkylation [25], *N*-acylation [26,27], and *N*-carboxyalkylation [28]. The solubility of chitosan in common organic solvents is enhanced by these chemical modifications, which also improve the properties of chitosan membranes [29].

We have successfully synthesized *N*-phthaloylated chitosan. This modification significantly enhanced the solubility of chitosan, as well as improved other properties, including film formability, flexibility, and controlled permeability [30]. The compactness of materials for coating membranes for controlled release fertilizers is another important property [31,32]. Even though *N*-phthaloylation of chitosan improved the compactness, mechanical strength and the biodegradability [30], further improvement to the controlled-release properties and mechanical strength was still needed to satisfy the stringent requirements for controlled release fertilizers.

Crosslinking is a common way to improve the controlled-release properties and mechanical strength by introducing a three-dimensional network structure [33]. Consequently, the motion of solutes across crosslinked polymer membranes can be controlled by precisely controlling this network structure. Therefore, we proposed the synthesis of crosslinked *N*-phthaloylated chitosan by crosslinking with suberoyl chloride. This long chain crosslinker not only introduces three-dimensional networks into the polymer, but also imparts significant flexibility to the obtained chitosan membrane. We also investigated the influences of different degrees of crosslinking of chitosan membranes on properties [such as hydrophobicity, mechanical strength, and permeabilities of plant macroelements (N, P, K), microelements (zinc, copper), and plant growth regulator (naphthylacetic acid, NAA)]. We anticipated that crosslinking would improve the compactness, mechanical strength, controlled permeability and hydrophobicity, whilst retaining the film formability and flexibility of the original *N*-phthaloyl acetylated chitosan.

## 2. Results and Discussion

### 2.1. Chemical Structure

**Figure 1:**
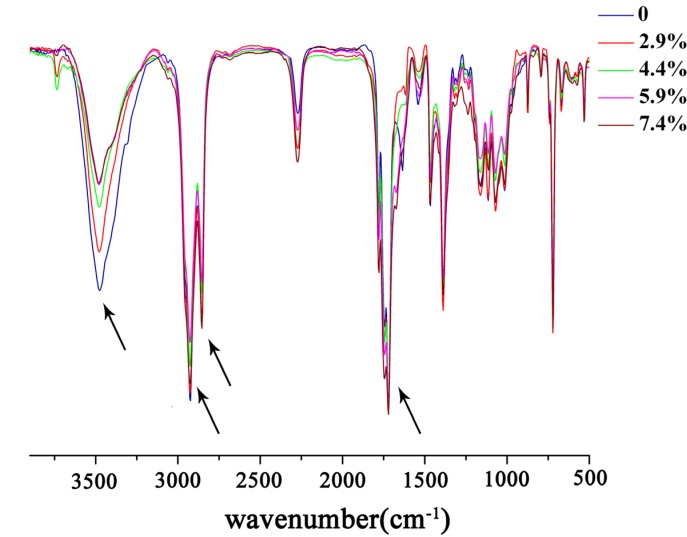
FT-IR spectra of *N*-phthaloyl acylated chitosan with different crosslinking densities.

The FT-IR spectra of *N*-phthaloyl acylated chitosan with different crosslinking densities are shown in Figure 1. The characteristic absorptions of chitosan, *N*-phthaloyl chitosan, and *N*-phthaloyl acylate chitosan were discussed in our earlier work [30]. 

**Figure 2:**
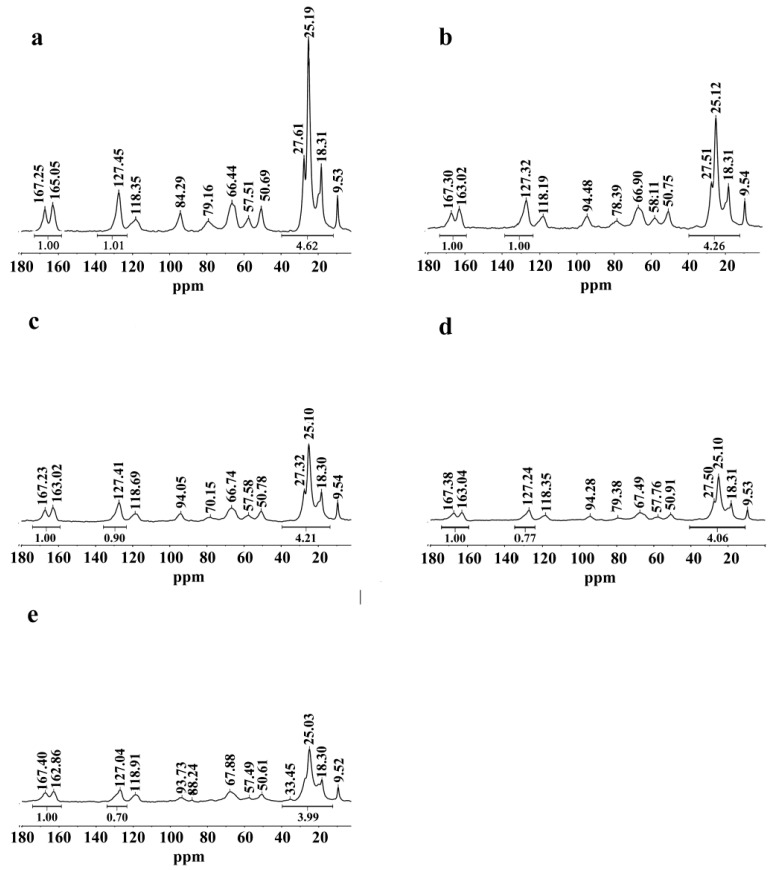
^13^C-NMR spectra of *N*-phthaloyl acylated chitosan with different crosslinking densities (**a**) 7.4%, (**b**) 5.9%, (**c**) 4.4%, (**d**) 2.9%, and (**e**) 0%.

Typical chitosan absorptions of were observed at 3,448 cm^−1^, 1,659 cm^−1^, 1,599 cm^−1^ and 1,321 cm^−1^. The broad band around 3,448 cm^−1^ was assigned to the stretching vibration of the inter- and intra- molecular hydrogen bonds from the −NH_2_ and −OH groups [30,34,35]. The bands at 1,659 cm^−1^, 1,599 cm^−1^ and 1,321 cm^−1^ were attributable to the peaks of stretching vibrations of amide C=O, N−H, and the peak of stretching and bending vibrations of C−N, respectively [36]. Compared with the chitosan spectra, peaks of the *N*-phthaloyl group appeared at 1,776 cm^−1^ and 1,712 cm^−1^; these are characteristic of the imide C=O stretching vibration. Another prominent peak at 721 cm^−1^ was assigned to the bending vibrations of –CH_2_ groups in the phthalate ring [37], and the peak at 1,747 cm^−1^ was attributed to the bending vibration of the ester groups (C=O). Other peaks at 2,929 cm^−1^ and 2,857 cm^−1^ corresponded to the asymmetric and symmetric bending vibrations of the methylene groups following the introduction of long chains in the acylation reaction [38,39]. After the crosslinking reaction, the intensity of the peak at 3,448 cm^−1^ was observed to weaken and diminished further with increasing amount of crosslinking agent, suggesting that the –OH groups had been transformed into the corresponding acetyl esters. The peaks at 1,747 cm^−1^, 2,929 cm^−1^, and 2,857 cm^−1^ were all enhanced slightly with the introduction of long chains in the crosslinking reaction. In conclusion, crosslinked *N*-phthaloyl acylated chitosan had been successfully prepared. To further validate the structure, the solid-state ^13^C-NMR spectra of *N*-phthaloyl acylated chitosan with different crosslinking degrees were obtained (Figure 2). In the TOSS mode, chitosan showed typical chemical shifts between 20 ppm to 100 ppm [40]. Compared with chitosan, new signals from the C=O in *N*-phthaloyl acylate chitosan were observed at 127.45 ppm, 163.05 ppm, and 167.25 ppm [25,41], and the new peaks from 10 ppm to 40 ppm are the signals from –CH_2_ and –CH_3_ [42]. From Figure 2, the peaks of C=O, –CH_2_, and –CH_3_ increased in intensity as more crosslinking agent was added. The integrated intensities of C=O, the order from the uncrosslinked sample to the sample with highest crosslinking density, were 1.70, 1.77, 1.90, 2.00, and 2.01. This is further evidence that the crosslinking reaction between *N*-phthaloyl acylate chitosan and suberoyl chloride was conducted successfully. The integrated intensities of –CH_2_ and –CH_3_, in order from the uncrosslinked sample to the sample with highest crosslinking density, were 3.99, 4.06, 4.21, 4.26, and 4.62, which is also consistent with successful crosslinking reaction.

### 2.2. Measurement of Hydrophobicity

**Figure 3:**
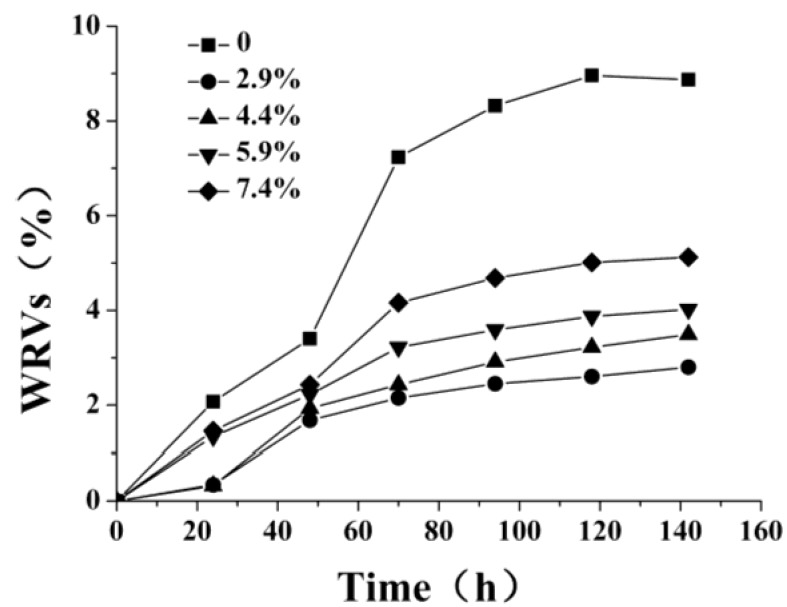
WRVs of *N*-phthaloyl acylated chitosan membranes with different crosslinking densities.

The hydrophobicity is an important parameter used to characterize the surface properties of membranes. Water retention values were used to evaluate the hydrophobicity, and were determined by the method described by Ioannis [43] and Siroka [44]. Figure 3 shows WRVs of the *N*-phthaloyl acylated chitosan membranes with different crosslinking degrees. The WRVs of *N*-phthaloyl acylated chitosan have already been greatly reduced (compared with that of chitosan) by the acylation reaction [30]. After crosslinking, the saturated water absorptivity was greatly reduced further, while the saturation time for all the membranes remained the same. The WRV profiles decreased with increasing crosslinking density increase, although only a very small amount of the suberoyl chloride crosslinking agent was added. Because the WRVs reflect the swelling degree of the membranes in water, and are influenced by the content of hydroxyl groups [45], this is further evidence that crosslinking has consumed the hydroxyl groups within the original polymer. After crosslinking, the malleability of the membrane has improved, as we expected.

### 2.3. Mechanical Properties

**Figure 4:**
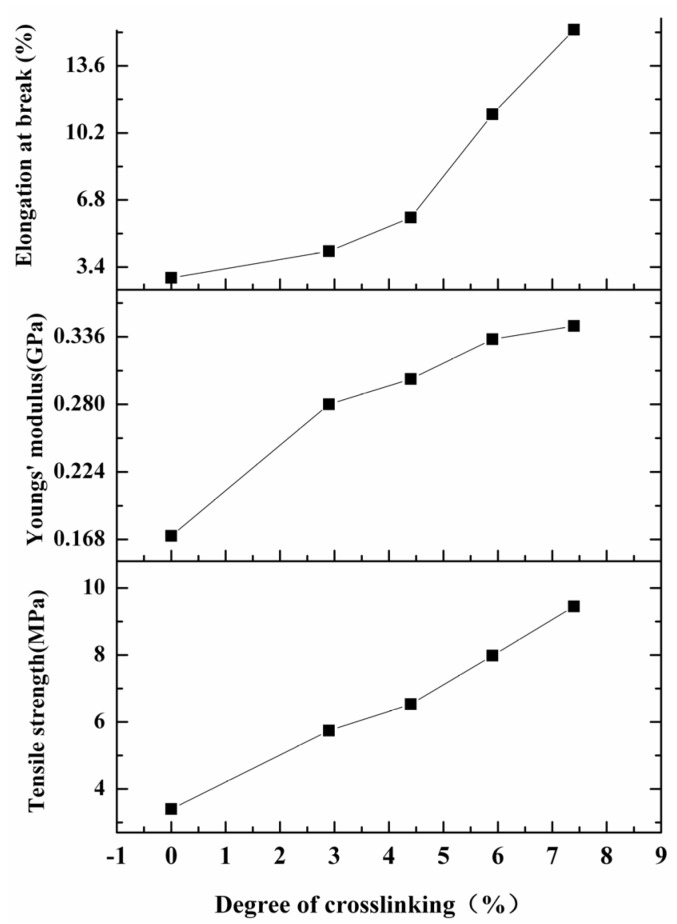
Mechanical properties of *N*-phthaloyl acylated chitosan with different crosslinking densities.

Good mechanical properties are important for membranes intended for use in controlled release fertilizers. Figure 4 shows the mechanical properties of *N*-phthaloyl acylated chitosan membranes with different degrees of crosslinking. Good mechanical behavior of the membranes, including increases in tensile strength, Young’s modulus, and elongation at break, was found; the properties increased consistently with increasing crosslinking density. These results arise from the formation of a three-dimensional network structure by crosslinking [33]. The increased extent of hydrophobic interactions may also contribute to the increase in mechanical properties, because longer side chains and a higher degree of substitution can stabilize the structure [46]. Surprisingly, the elongation at break was also improved together with the tensile strength and Young’s modulus, indicating that crosslinking *N*-phthaloyl acylated chitosan membranes a far superior combination of mechanical properties for further applications. The variation in mechanical properties with crosslinking density suggests that the mechanical properties of the crosslinked membranes can be tuned by regulating the crosslinking density.

### 2.4. Permeability of Macro-Nutrients and Micro-Nutrients

**Figure 5:**
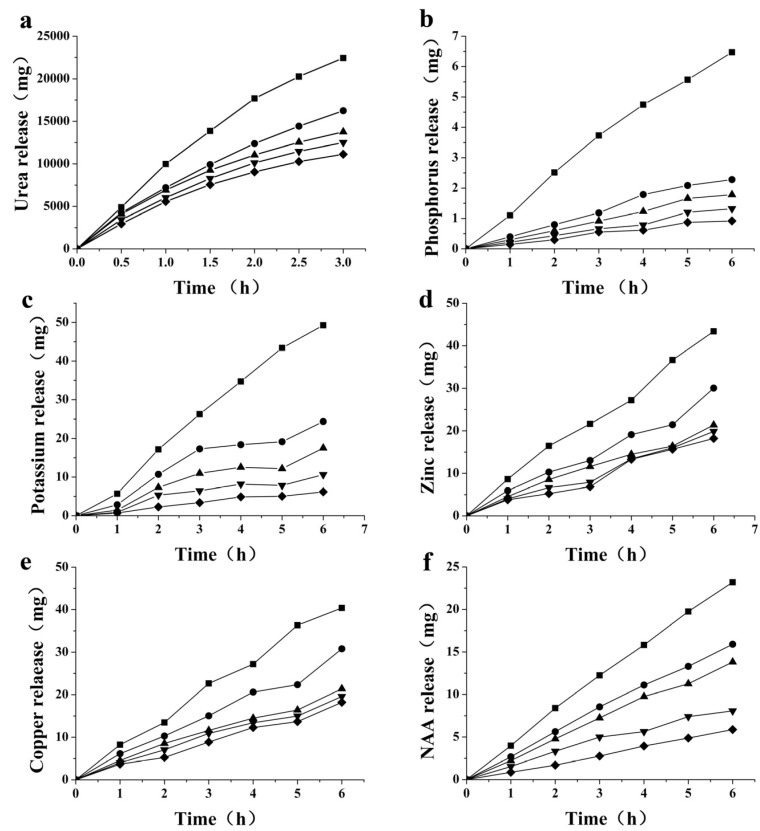
(**a**) urea; (**b**) phosphorus; (**c**) potassium; (**d**) zinc; (**e**) copper; (**f**) NAA; permeabilities of *N*-phthaloyl acylated chitosan with different crosslinking densities ■ 0%; ● 2.9%; ▲ 4.4%; ▼ 5.9%; ◆ 7.4%.

Urea and K_2_HPO_4_ are the most basic nitrogen, phosphate and potash fertilizers used in agricultural production. Excessive macro-nutrients can lead to seedlings being “burnt” by fertilizer, and also result in environmental pollution, so controlled release of N/P/K is necessary. Permeability of N/P/K is one of the most important parameters when evaluating the controlled-release properties of a membrane coating for a fertilizer. We used urea and K_2_HPO_4_ to do this, because of their widespread role in agricultural production. The accumulated penetration of all matter across a crosslinked *N*-phthaloyl acylated chitosan membrane is shown in Figure 5. All curves revealed a time-dependent release pattern. Figure 5a shows the accumulated urea penetration of *N*-phthaloyl acylated chitosan membranes with different crosslinking degrees. The overall amount of urea released was remarkably reduced compared with the release from *N*-phthaloyl acylated chitosan without crosslinking, and the permeability dropped further with increased crosslinking. This is expected, because enhanced crosslinking density decreases the free volume within the network structure. However, as the crosslinking density increases past a certain stage, further increases in amount of suberoyl chloride lead to poor film-forming ability, and consequently poor permeability. Figure 5b,c show phosphorus and potassium accumulated penetration profiles, and all show the same features as seen for the urea permeability. The data suggested that the crosslinking reaction improved the densification and hydrophobicity of the membranes; these changes are highly desirable for application in controlled-release fertilizers.

Microelements such as zinc, copper, manganese are essential nutrients for plants, and microelement-containing fertilizers are become more and more popular. Not only does zinc deficit reduces the yield and production of crops, it also results in reduction of their nutritional value. In addition to having an important role in activating the enzymatic systems of plants, zinc is essential for synthesis of chlorophyll and carbohydrates [47]. Copper deficit is a major problem with most limey soils; copper plays an important role in increasing agricultural yield. Figure 5d,e present the permeability of zinc and copper ions. The permeability of both microelements decreased with increasing amount of crosslinking, as expected from the previous results. These results clearly demonstrate that membranes can be used in microelement fertilizers.

### 2.5. Permeability of Plant Growth Regulator NAA

1-Naphthylacetic acid (NAA) is a well-known plant growth regulator (PGR), which belongs to the auxin family [40]. It is a rooting agent, and is used for the vegetative propagation of plants from stem and leaf cuttings. Figure 5f shows the accumulated penetrating mass of NAA across *N*-phthaloyl acylated chitosan membranes with different crosslinking degrees. The results demonstrate that relatively large molecules are able to permeate across the membrane. As expected, the permeability decreased as crosslinking increased. The masses of solutes moved in Figure 5 can be ranked in the following descending order: urea > K^+^ > Zn^2+^ ≈ Cu^2+^ > NAA > HPO^4−^; one possible explanation for this order is that the higher the solubility of the nutrient, the higher the amount of nutrients which can be released from the membranes [48].

## 3. Experimental

### 3.1. Materials

Chitosan derived from crab shells (molecular weight 5,000) was purchased from Gold-shell Biochemical Co., Ltd. (Taizhou, China). The degree of deacetylation (as determined by ^1^H-NMR) was 90%, and the chitosan was used without further purification. Dodecanoyl chloride and suberoyl chloride were purchased from Tokyo Chemical Industry Co., Ltd. (Tokyo, Japan) and Sigma (Shanghai, China), respectively, and were used as received. All other chemicals were purchased from Sinopharm Chemical Reagent Co., Ltd. (Shanghai, China). *N,N*-dimethylformamide (DMF) was stirred with CaH_2_ (50 g/L) overnight, followed by vacuum distillation at 20 mmHg, prior to use. Analytical grade phthalic anhydride was used without further purification.

### 3.2. Synthesis and Film Forming of Crosslinked N-Phthaloyl Acylated Chitosan

**Scheme 1:**
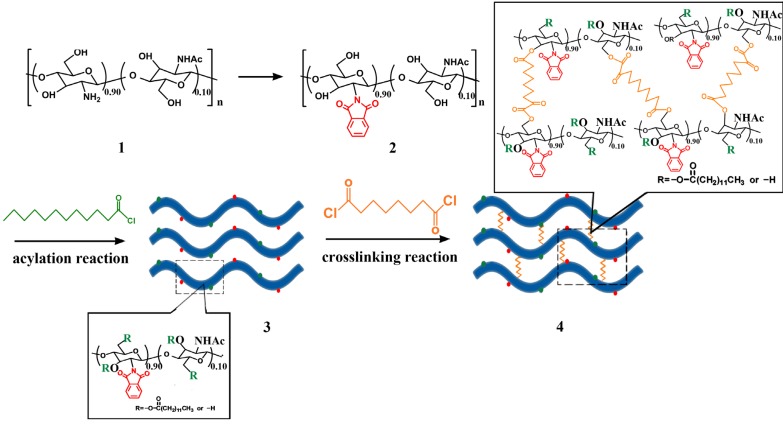
Synthetic route for N-phthaloyl acylated chitosan and crosslinked *N*-phthaloyl acylated chitosan.

The synthetic procedure used for the preparation of crosslinked *N*-phthaloyl acylated chitosan is depicted in Scheme 1; the synthetic procedure for *N*-phthaloyl acylated chitosan precursor was detailed in our previous work [30]. Briefly, chitosan (1.0 g) was reacted with phthalic anhydride (2.76 g) in a mixture of DMF and distilled water (20 mL, 95:5 v/v) at 125 °C under nitrogen for 8 h. *N*-phthaloylated chitosan was obtained after three cycles of washing with methanol and filtration. Subsequently, *N*-phthaloylated chitosan (2.0 g) was reacted with dodecanoyl chloride (6.03 mmol) in a mixture of DMF and pyridine (2:1) for 6 h at room temperature. The mixture was poured into a mixture of iced water and methanol (2:1) and the precipitated product was then collected by filtration. *N*-phthaloyl acylated chitosan was obtained after three cycles of precipitation and filtration. 

*Reagents and conditions*: (1) chitosan; (2) phthalic anhydride, DMF/water, 120 °C, 8 h; (3) dodecanoyl chloride, DMF/pyridine, 6 h; (4) suberoyl chloride, DMF/pyridine, 6 h.

Prepared *N*-phthaloyl acylated chitosan (2.0 g) was dissolved in a mixture of DMF (30 mL) and pyridine (15 mL) and stirred under nitrogen at room temperature. The mixture was then cooled in an ice-salt bath and 80 μL suberoyl chloride was added drop-wise. The resultant mixture was stirred for 6 h at room temperature, and then poured into a bath of iced water and methanol (300 mL, 2:1 v/v); a yellowish flocculent precipitate emerged. The resulting precipitated product was collected by filtration, and was then suspended in methanol (300 mL), stirred at room temperature for 4 h and collected by filtration again. The crosslinking density was stated as 2.9%, according to the amount of added crosslinker compared to the amount of modified chitosan.

The product was dissolved in ethyl acetate and poured onto a tetrafluoroethylene plate, and then dried under vacuum at 40 °C overnight to give the desired crosslinked *N*-phthaloyl acylated chitosan membrane. *N*-phthaloyl acylated chitosans with different crosslinking degrees were synthesized with the same method, except that different amounts (120, 160, or 200 μL) of suberoyl chloride were added, and the crosslinking densities were stated as 4.4%, 5.9%, and 7.4%, respectively.

### 3.3. FT-IR Spectroscopy

FT-IR spectra were recorded on a Nicolet NEXUS-470 Spectrometer from Thermo Fisher Scientific (Madison, WI, USA) from KBr pellets at room temperature. Samples (2 mg) were thoroughly ground with KBr and pellets were prepared using a hydraulic press under a pressure of 600 kg/cm^2^. All spectra were recorded with an accumulation number of 32 scans and a resolution of 8 cm^–1^.

### 3.4. Solid-State ^13^C-NMR

Solid-state ^13^C Cross-Polarization Magic Angle Spinning (CP/MAS) NMR spectra were taken on a BRUKER DMX 400 instrument (Bruker, Bremen, Germany) at 25 °C using a 7 mm probe with a ^13^C frequency of 100.4 MHz, 90 deg pulse, 4.17 µsec, 101 Watt using either Total Suppression of Sidebands (TOSS) or TOSS with Dipolar-dephasing for Non-quaternary Carbon Suppression (TOSDL-NQS) modes.

### 3.5. Mechanical Properties

The crosslinked *N*-phthaloyl acylated chitosan films were cut into dumbbell-shaped samples with gauge length 15 mm, width 5 mm and thickness of 60 μm. Four tests were performed at room temperature on each of the films, using an electronic universal testing machine C43 (Shenzhen, China) at a stretching speed of 2 mm/min.

### 3.6. Water Retention Values (WRVs)

For water retention value determination, the chitosan and crosslinked *N*-phthaloyl acylated chitosan membranes with different DS values were cut into small pieces of equal size and weighed. The pieces were subsequently soaked in distilled water for 7 days and removed for weighing every 24 h during this time. The surface water was carefully wiped off with tissue paper prior to weighing. All procedures were conducted at room temperature and the *WRVs* were calculated as the amount of absorbed water per unit dry film mass (Equation 1).

*WRVs* (%) = (*W_t_* − *W_0_*)/*W_0_* × 100 (1)

where *W_t_* is the total weight of the membrane at time *t* and *W_0_* is the initial mass of the membrane. Each test was performed in triplicate with the results subsequently being averaged.

### 3.7. Permeability of N/P/K, NAA, Cu^2+^ and Zn^2+^

**Figure 6:**
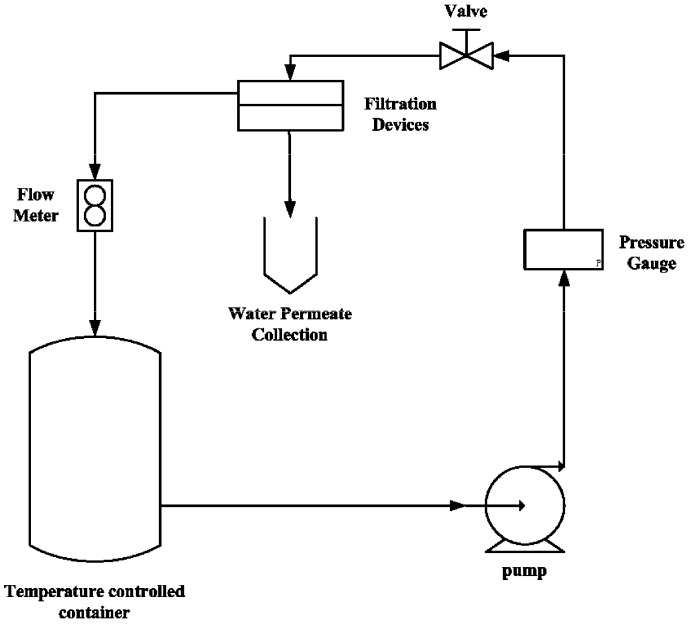
Schematic diagram of self-built temperature controlled equipment.

The permeability tests were performed at 0.75 MPa on a customized temperature controlled apparatus (Figure 6). Initially, each membrane was immersed in distilled water for at least 24 h. The effective membrane area was around 4 cm^2^ and the tests were carried out a duration of at least 6 h. K_2_HPO_4_ (25 g/L) was used as source of P and K, and added into the temperature controlled tank (25 °C); the permeated solution was collected from the gathering unit every hour. The concentration of P was measured by a UV spectrometer at a wavelength of 450 nm after to the chromogenic reaction of P with ammonium vanadomolybdate [49]. The concentration of K was determined by the potassium-TPB gravimetric method [50,51].

The permeability of N (urea) was measured using a similar procedure to that described above, with urea (25 g/L) in the container. The transmitted urea concentration was determined at a wavelength of 426 nm after chromogenic reaction of urea with *p*-dimethylaminobenzaldehyde [30,52]. All tests were performed in triplicate with the average results being reported.

The permeability of NAA was using a similar procedure, but at 50 °C. The initial NAA concentration was 25 g/L, and the permeated NAA concentration was analyzed by UV at 281 nm.

The permeability of microelements was measured at 50 °C, with ZnSO_4_ (25 g/L) and CuSO_4_ (25 g/L) solution in the container. The permeated zinc and copper were determined by EDTA titrations, for which the pH values were controlled within the range 6–8 and 10–11, respectively.

## 4. Conclusions

Crosslinked *N*-phthaloyl acylated chitosan membranes have been synthesized. The hydrophobicity and mechanical properties were greatly improved, even though only a small amount of crosslinking agent was included. Decreases in the permeability of N/P/K, NAA, and microelements were observed upon crosslinking. These reductions in permeability make the materials appropriate for use in controlled-release microelement fertilizers. Most importantly, the permeability of the membrane could be optimized by adjusting the amount of crosslinking agent used. This ability to tailor the membranes means that there is significant potential for application of these materials in agrochemicals.
